# Small Molecule with Big Impact: Metarrestin Targets the Perinucleolar Compartment in Cancer Metastasis

**DOI:** 10.3390/cells13242053

**Published:** 2024-12-12

**Authors:** Vivek K. Kashyap, Bhuvnesh P. Sharma, Divya Pandey, Ajay K. Singh, Godwin Peasah-Darkwah, Bhupesh Singh, Kuldeep K. Roy, Murali M. Yallapu, Subhash C. Chauhan

**Affiliations:** 1 Division of Cancer Immunology and Microbiology, Medicine, and Oncology Integrated Service Unit, School of Medicine, University of Texas Rio Grande Valley, McAllen, TX 78504, USA; 2 South Texas Center of Excellence in Cancer Research (ST-CECR), McAllen, TX 78504, USA; 3 Department of Biotechnology, Bhagwant University, Ajmer 305004, Rajasthan, India; 4 Department of Pharmaceutical Sciences, School of Health Sciences and Technology, UPES, Dehradun 248007, Uttarakhand, India; 5 Department of Oncology, Albert Einstein College of Medicine, Bronx, NY 10461, USA; 6 School of Applied Sciences, OM Sterling Global University, Hisar 125001, Haryana, India

**Keywords:** Perinucleolar compartment (PNC), RNA polymerase III transcripts, metarrestin, cancer metastasis, eEF1A1, eEF1A2, pharmacokinetic/toxic properties (ADME/T)

## Abstract

Metarrestin (ML246) is a first-in-class pyrrole–pyrimidine-derived small molecule that selectively targets the perinucleolar compartment (PNC). PNC is a distinct subnuclear structure predominantly found in solid tumor cells. The occurrence of PNC demonstrates a positive correlation with malignancy, serving as an indicator of tumor aggressiveness, progression, and metastasis. Various promising preclinical results have led to the clinical translation of metarrestin into a first-in-human trial. This review aims to summarize (i) the current understanding of the structure and function of PNC and its role in cancer progression and metastasis, (ii) key findings from studies examining the effect of metarrestin on various cancers across the translational spectrum, including in vitro, in vivo, and human clinical trial studies, and (iii) the pharmaceutical relevance of metarrestin as a promising anticancer candidate. Furthermore, our molecular docking and MD simulation studies show that metarrestin binds to eEF1A1 and eEF1A2 with a strong and stable affinity and inhibits eEF1A2 more efficiently compared to eEF1A1. The promising results from preclinical studies suggest that metarrestin has the potential to revolutionize the treatment of cancer, heralding a paradigm shift in its therapeutic management.

## 1. Introduction

Cancer is a major public health problem worldwide and is the second leading cause of death in the United States [1]. Malignant tumors are characterized by uncontrolled cellular proliferation and the potential to evade surrounding tissues [2]. These tumors may spread to other regions of the body through a process known as metastasis [2]. Metastasis is a multistage and intricate process by which cancer cells escape primary tumors, infiltrate vascular and lymphatic systems, and colonize into distant organs of a new and different microenvironment [2,3]. Metastatic colonization is governed by well-characterized cellular processes and signaling pathways [3,4,5,6,7,8,9,10,11]. These include epithelial-to-mesenchymal transition (EMT), up-regulation of the interleukin-6 (IL-6)-mediated signal transducer and activator of transcription 3 (STAT3), and activation of the MAPK, Wnt/β-catenin, PI3K-AKT-mTOR, and JAK/STAT signaling cascades [4,5,6,7,8,9]. Additionally, transcriptional programs driven by snail family transcriptional repressor 1 (SNAI1), dysregulation of exosome biogenesis, and the expression of various types of non-coding RNAs play pivotal roles [12,13,14]. A high prevalence of PNC has been implicated in the metastasis process [10,11,15,16]. Despite significant advancements in cancer detection and therapeutic strategies, metastasis remains the primary cause of cancer-related mortalities [17,18]. Metastasis cancer treatment is hindered by the disease’s complexity and incomplete understanding, including treatments focusing on a single gene or gene product less likely to be successful [18,19].

A new cellular phenotypic biomarker has been recently identified, known as the perinucleolar compartment (PNC), which is highly prevalent in metastatic cancer cells [16]. The PNC is a subnuclear structure predominantly found in cancer cells and is absent in normal cells, including embryonic stem cells [15]. The PNC is highly enriched with small nuclear RNAs and RNA-binding proteins [20,21], associated with chromatin [22], involved in RNA metabolism and Pol III function [22,23,24,25], and strongly associated with metastatic potential and cancer progression [26]. The prevalence of PNC is a highly reliable indicator for both prognosis and prediction of the overall clinical outcome, including survival [15,16,27]. Thus, the PNC is a key marker for metastatic cancer for the development of therapeutics [28].

**Figure 1:**
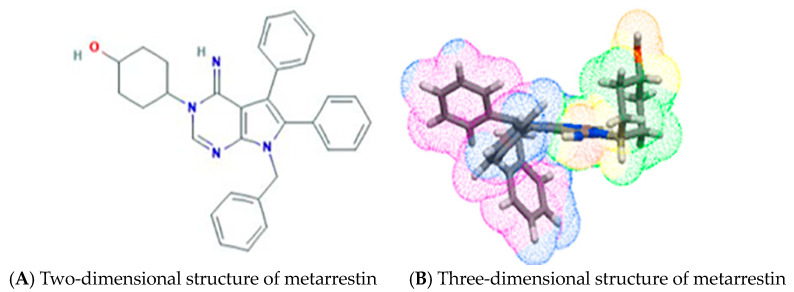
Two- and three-dimensional chemical structure illustrations of metarrestin. (**A**) The 2D chemical structure of metarrestin was downloaded from PubChem [31]. (**B**) The 3D chemical structure of metarrestin was downloaded from a free accessible online service, Molinspiration Galaxy 3D Structure Generator v2023.08 (https://www.molinspiration.com/cgi/galaxy, Slovensky Grob, Slovakia, accessed on 30 November 2024).

Metarrestin (ML246) represents a first-in-class small-molecule inhibitor specifically designed to disrupt the PNC within cancer cells (Figure 1) and inhibits migration and invasion in various cancer cell lines [26]. The intriguing and novel mechanism of action has been reinforced by an encouraging pharmacological profile of the molecule, as examined in rodents and Ras-driven autochthonous KPC (Pdx1-Cre;LSL-Kras^G12D/+^;Tp53^R172H/+^) mice [29]. Preliminary pharmacokinetic (PK) studies indicate that metarrestin is a low-clearance drug with high tissue/plasma area under the curve (AUC)_0-24 hrs_ ratios in multiple organs [30]. This review article provides a comprehensive overview and analysis of the PNC in malignancy, focusing on metarrestin’s chemical characteristics, anticancer potential, toxicity, pharmacological significance, and novel molecular mechanisms of action. This review aims to offer valuable insights and a basis for clinical studies in the development of metarrestin as a potential anticancer drug candidate.

## 2. PNC Drives Cancer Development, Progression, and Drug Resistance

PNC is a nuclear substructure that is not confined by a membrane and is distinct from the nucleolus. It has an irregular form and a size range of 0.25 to 4 microns. Electron microscopy studies have shown that the PNC is structured like a reticulated meshwork on the nucleolus surface, with electron-dense strands ranging from 80 to 180 nanometers (nm) in length [32,33].

The PNC is primarily detected in solid tissue cancer cells or cell lines derived from patient tumors or transformed in vitro, and it is extremely rare in normal cells, such as human or mouse embryonic stem cells [16,32]. Studies indicate that the PNC structure is preserved throughout the interphase of the cell cycle, disassembles during mitosis alongside the nucleoli, and reassembles during late telophase when nucleoli reform [32]. The prevalence of the PNC varies across cancer types and transformed cell lines, demonstrating a positive correlation with in vivo tumor aggressiveness and cancer progression [34,35]. Notably, elevated PNC prevalence has been observed in aggressive regions of primary breast, ovarian, and colon tumors [15,16,27,32]. While PNC prevalence in primary tumors exhibits variability, it significantly increases in metastatic lesions, often reaching 100% in distant metastases. The strong relationship between PNC occurrence and malignancy in vitro and in vivo suggests that PNC production represents a crucial transformation process that signals cancer cell spreading capacity.

Furthermore, ideal tumor markers should be those associated with the pathophysiology of cancer cells (unlimited anchorage-independent growth and metastasis), and their reduction should indicate improved cellular behavior and better patient outcome [36]. Such a marker may reflect malignant activity. PNC prevalence may serve as a malignancy-specific phenotypic marker to screen for anticancer compounds that disrupt processes crucial for cancer cell survival [37].

**Figure 2:**
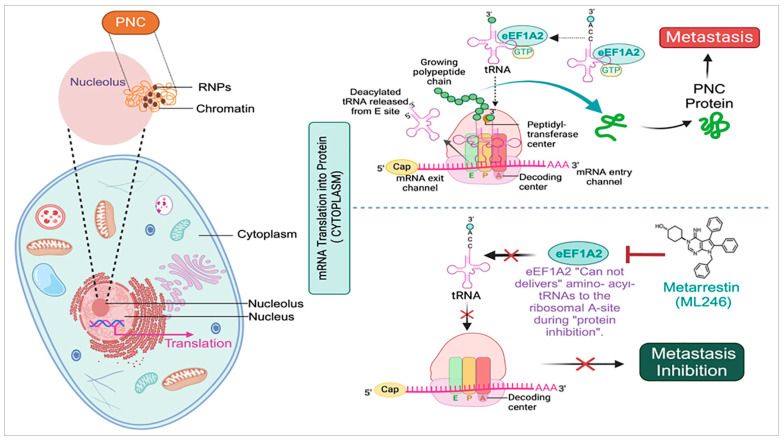
The molecular mechanism of metarrestin. The PNC consists of dynamic structures located in the proximity of nucleolus within the cell. These compartments consist of RNA-binding proteins that play a crucial role in various biological processes by storing and regulating RNA molecules (transcription). Additionally, PNC influences gene expression in response to different cellular signals, highlighting their importance in cellular function and regulation. During protein synthesis (translation), eEF1A2 binds to tRNA to deliver aminoacyl-tRNA to the ribosomal A-site for the elongation of polypeptide chains. This process maintains the structural integrity of the PNC, which is responsible for causing metastasis (upper panel). Metarrestin inhibits the transfer of aminoacyl to the ribosomal A-site, which is crucial for the growth of the polypeptide chain. As a result, it suppresses the protein synthesis involved in metastasis, leading to metastasis inhibition (lower panel). This image was created with BioRender.com on 30 November 2024 (BioRender.com/y69o062).

Mechanistically, PNC is involved in the formation of metastatically capable cancer cells. The development of PNC is not related to normal and cancer cell growth characteristics, such as proliferation rate, glycolysis, and differentiation status [16] (Figure 2). PNC inhibitors actively disrupt cellular mechanisms that maintain malignancy, resulting in PNC loss. A metastatic prostate cancer cell line, PC3M, was engineered to stably produce GFP-fused polypyrimidine-tract-binding protein (PTB). The cell line has a PNC prevalence of 75% to 85% [32]. PTB are RNA-binding proteins (RBPs) with diverse activities, including regulating alternative splicing (AS), selecting polyadenylation sites, maintaining mRNA stability, and facilitating internal ribosome entry site (IRES)-mediated translation [38,39,40]. PTB are encoded by a family of highly conserved paralogous genes, with PTBP1 expressed in various cell types and PTBP2 and PTBP3 (previously ROD1) predominantly expressed in neurons and hematopoietic cells [41,42]. Studies reveal that PTBP1 is abundantly expressed and contributes to the malignant biological activity of bladder, colon, and breast cancer cells [39,43,44,45,46]. PTB serves as crucial markers of the PNC and are typically assessed through immunohistochemistry [47]. The self-reporting fluorescent PC3M-GFP-PTB cell line provides a sensitive and robust platform for image-based high-throughput, high-content assays (HCAs) [28]. This system facilitates the identification of test compounds or drugs capable of reducing PNC prevalence by at least 50%.

The PNC is increasingly linked to cancer; however, its molecular function and involvement in cancer are not well understood [37]. PNC localization studies show that certain non-coding Pol III RNAs and RNA-binding proteins involved in pre-mRNA processing are enriched [20,21,23]. The PNC was first identified during the analysis of the PTB [48]. In addition, PTB is a protein with a high degree of dynamism that shuttles between the nucleus and the cytoplasm [49], and this process is regulated by phosphorylation [50]. PTB exhibits multifunctionality and is believed to engage in both nuclear and cytoplasmic processes by binding to pyrimidine-rich RNA sequences and serving as an intermediary, connecting RNAs with various cellular components that perform distinct activities [49]. Many of these activities are altered in cancer cells [51,52].

Several additional RNA-binding proteins, such as CUGBP, KSRP, Raver1, and Raver2, are localized within the PNC alongside PTB [53,54,55,56]. The PTB-associated splicing factor (PSF) and p54nrb, involved in RNA editing and processing, are also associated with the PNC; however, the mechanism governing RNA binding within the PNC remains unclear [57,58]. Studies demonstrate that PTB’s localization to the PNC is determined by its RNA-binding ability. While inhibition of RNA polymerase II activity does not alter PTB’s immunolabeling within the PNC, treatment with RNase A significantly diminishes its presence, highlighting the dependence on RNA for PTB association [32]. Evidence suggests that the PTB’s role in the PNC is linked to RNA-binding function, though it may not directly influence the pre-mRNA process. Polloc et al. demonstrated that the protein PTB interacts directly with small RNA molecules transcribed by RNA polymerase III within the PNC in an in vivo model [24]. Super-resolution microscopy analysis reveals the co-localization of PTB and a specific small RNA known as RNase MRP RNA in the PNC, forming a reticulated meshwork [24]. Importantly, knockdown of PTB significantly reduces the frequency of PNC structures, suggesting its vital role in the proper localization of PNC components like RNase MRP RNA and CUGBP to the PNC [23,24]. CUGBP, or CUG triplet repeat-binding protein, is found mostly in the nucleoplasm and is abundant in the PNC. CUGBP regulates alternative splicing, C-to-U RNA editing, deadenylation, mRNA decay, and translation [53,59,60]. CUGBP1 overexpression is an independent indicator of poorer survival post-surgery and is correlated with the TNM stage in non-small cell lung cancer [61]. KSRP (KH-type splicing regulatory protein) is a multidomain RNA-binding protein that is involved in a number of biological activities, including nucleus splicing and cytoplasmic mRNA localization [62,63]. KSRP is predominantly found within the nucleus of a cell, and its localization pattern closely resembles that of polypyrimidine-tract-binding protein. The presence of KSRP is found to be more prominent in the PNC of neuroblastoma cells than in HeLa cells [54]. Raver1 is an 80 kDa ribonucleoprotein with 756 amino acids and three RRMs. Raver1 is located on chromosome 19p13.2 [64,65]. RAVER1/2 directly interact with PTB to influence and regulate alternative splicing events within a cell [56]. ROD1 exhibits similarity with PTB and is a homologous counterpart to the yeast regulator of differentiation [66].

Additionally, the PNC is also enriched with several small RNAs transcribed by RNA polymerase III, specifically including RNase MRP and RNase P RNA, SRP RNA, Alu repetitive elements, and certain types of hY RNAs associated with the Ro autoantigen [20,21,23]. RNase MRP RNA and RNase P H1 RNA are conserved sequence-specific endoribonucleases. They have structural similarities [67,68,69,70] and the ability to bind shared protein subunits, resulting in the formation of functional RNase particles [67]. RNase MRP plays a vital function in the processing of pre-rRNA and the replication of mitochondrial DNA [71,72,73], whereas RNase P is involved in the processing of tRNA and pre-rRNA [67,74]. The hY RNAs are small non-coding RNA molecules that specifically bind to Ro proteins, resulting in the formation of Ro ribonucleoproteins (RNPs), where “hY” stands for “human Y” RNA, referring to their structural characteristics [75]. While the exact function of Ro RNPs is still unknown, they are considered to have a role in RNA stability, stress response, and possibly DNA replication [76]. Alu RNA molecules are organized into an Alu complex, which functions in controlling the processes of translation and transcription [77,78]. The signal recognition particle RNA (SRP RNA) component is a key part of the signal recognition particle (SRP), which plays a role in transporting secretory proteins and halting the elongation process to direct the protein toward the endoplasmic reticulum membrane for further processing and secretion [79].

Several studies have shown that the PNC is involved in RNA processing and metabolism within the cell [32,33,34]. PNC localization and integrity depend on the RNA-binding ability of PTB [32]. These data suggest that the PNC is involved in RNA metabolism. Multiple studies have demonstrated that the PNC is predominantly enriched with newly synthesized RNA transcribed by RNA polymerase III (Pol III), rather than the RNA polymerase I (Pol I) or II (Pol II) [23,80,81,82,83]. PNC nucleates on one or more DNA loci and establishes connections with chromatins [28]. PNC-associated loci replicate early in the mid-S phase, peaking 3 hrs after hydroxyurea release [28]. PNC-enriched RNAs, such as RNase MRP and RNase P RNA, are not transcribed within the PNC because their gene loci are not co-localized with it [20,21,24]. In PNC-containing cells, the nucleation of the RNA–protein complex on the DNA loci may directly regulate gene expression [24,28,32]. Overall, the PNC is being developed as a prognostic indicator for various types of cancer. The eradication of the PNC in cancer cells has demonstrated its potential as a screening technique for identifying probe chemicals, which could aid in investigating PNC biology and potentially lead to the development of less toxic chemotherapy drugs.

## 3. Identification of Metarrestin, a First-in-Class Small-Molecule Selective PNC Inhibitor

Metarrestin [Trans-4-(7-benzyl-4-imino-5,6-diphenyl-4,7-dihydro-3H-pyrrolo [2,3-d]pyrimidin-3-yl) cyclohexanol] was identified through a comprehensive drug screening of a diverse library of over 140,000 compounds. The compounds were selected based on their capacity to disassemble fluorescence labeled PNC structures, as determined by high-throughput immunofluorescence microscopy imaging [28,84]. The initial screening results were re-evaluated and reassessed to distinguish the disassembling activity of the PNC from the cytotoxic mechanism of DNA intercalators and topoisomerase I and II inhibitors. This was achieved through assays that measured cytotoxicity, apoptosis, or DNA intercalation displacement. After optimizing its medicinal chemistry, the compound metarrestin was identified as the most promising candidate [84]. Huang et al. showed that metarrestin disrupts PNC assembly by blocking RNA polymerase I transcription [26]. Metarrestin effectively disrupts PNCs in cancer cell lines at submicromolar concentrations, blocks metastatic development, and significantly prolongs survival in preclinical mouse models [84].

Furthermore, no toxicities associated with metarrestin have been observed in an in vivo mouse model [26,84]. Thermal stability and siRNA phenocopy studies have demonstrated that eukaryotic translation elongation factor 1 alpha 2 (eEF1A2) is a potential molecular target of metarrestin [26]. To identify metarrestin’s binding target, a biotin-conjugated probe was synthesized that effectively disassembled the PNC. The binding target of metarrestin was identified as eEF1A2 through affinity purification employing a biotin-conjugated probe and competition experiments with untagged metarrestin. Additionally, the interaction of metarrestin with eEF1A2 was confirmed through a cellular thermal shift assay. Research showed that eEF1A2 plays a role in enhancing the PNC assembly and the advancement of metastasis. Furthermore, it is believed that eEF1A2 partially mediates the PNC elimination function of metarrestin (Figure 2) [26]. While further information is needed, metarrestin may interfere with eEF1A2′s non-translational activities. A proteolysis-targeting chimera (PROTAC) approach was developed [85,86,87] which involved the tethering of metarrestin with different ligands to target the von Hippel–Lindau (VHL) E3 ligase [88]. Therefore, the goal of designing the acquired heterobifunctional molecules was to target eEF1A2 for selective destruction by the ubiquitin/proteasome system (UPS), since it is the binding target of metarrestin. Among these first-in-class eEF1A2 degraders, one showed potential for treating eEF1A2-mediated carcinogenesis since it degraded eEF1A2 (Figure 2).

## 4. Computational Elucidation of the Binding Mechanism of Metarrestin with eEF1A1 and eEF1A2 Through Molecular Docking and Molecular Dynamics (MD) Simulations

The eEF1A, previously known as eEF-1, is a crucial GTPase that has remained evolutionarily conserved throughout a wide variety of eukaryotes [89]. eEF1A1 and eEF1A2 are translation factors and pleiotropic proteins that exhibit elevated expression levels in human cancers, including breast, ovarian, and lung malignancies [90]. In addition to facilitating the enzymatic transport of aminoacyl-tRNAs to the ribosome, eEF1A1 has been documented to regulate cytoskeleton function, demonstrate chaperone-like properties, and regulate cell proliferation and apoptosis [90,91,92,93,94,95,96]. Conversely, eEF1A2 is highly expressed in solid organ cancers, including pancreatic cancer, as compared to normal tissues [97]. It is also overexpressed in metastatic sites such as lymph nodes and distant organs, in contrast to primary tumors [97]. Furthermore, elevated levels of eEF1A2 expression are linked to poor survival outcomes [98,99,100,101]. eEF1A2 is crucial for several fundamental cellular functions, including protein synthesis, maintenance of the cytoskeletal architecture, and regulation of nuclear export [102,103]. Additionally, it activates phospholipid signaling, Akt-dependent cell motility, and actin remodeling, all of which contribute to tumor progression [104,105,106,107,108].

**Figure 3:**
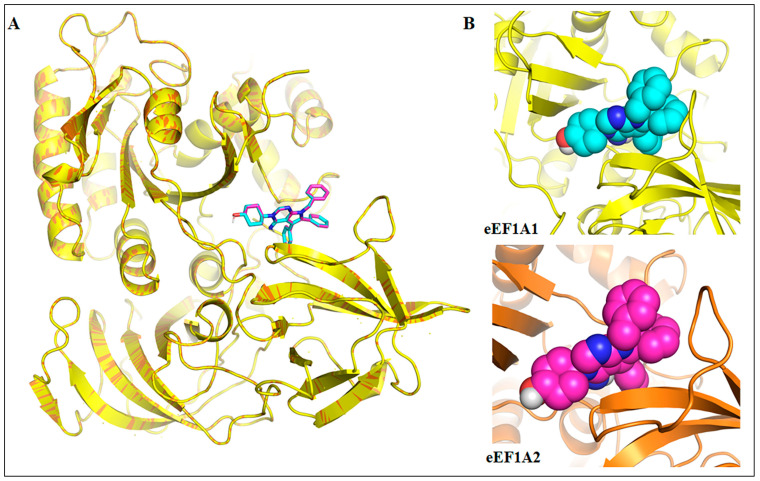
(**A**) Superimposition of eEF1A1 (yellow cartoon) and eEF1A2 (orange cartoon) with docked metarrestin (cyan and magenta sticks, respectively), and (**B**) Close views of the binding modes of metarrestin with eEF1A1 and eEF1A2. All the files of proteins and ligands were converted into PDBQT formats using AutoDock 4.2 [109,110,111,112]. Docking was carried out using the AutoDock Vina using the default settings, where blind docking was carried out initially using the whole protein to find the best suitable sites for metarrestin (Grid center X = 18.78, Y = 32.684, and Z = 30.586; Grid size 40 Å). Afterwards, considering the top solution, focused docking was performed at the best site (Grid center X = 39.648, Y = 39.314, and Z = 39.448; Grid size 20 Å).

To understand the binding characteristics of metarrestin with eEF1A1 and eEF1A2 proteins, molecular docking and MD simulations were carried out on the homology models of these proteins. Figure 3A shows the superimposition of eEF1A1 (yellow cartoon) and eEF1A2 (orange cartoon) with docked metarrestin (cyan and magenta sticks, respectively). Metarrestin was found to bind to the same conserved binding site of both the eEF1A1 (binding affinity: −9.0 kcal/mol) and eEF1A2 (binding affinity: −9.0 kcal/mol). Figure 3B depicts a close-up view of the binding modes of metarrestin with eEF1A1 and eEF1A2.

**Figure 4:**
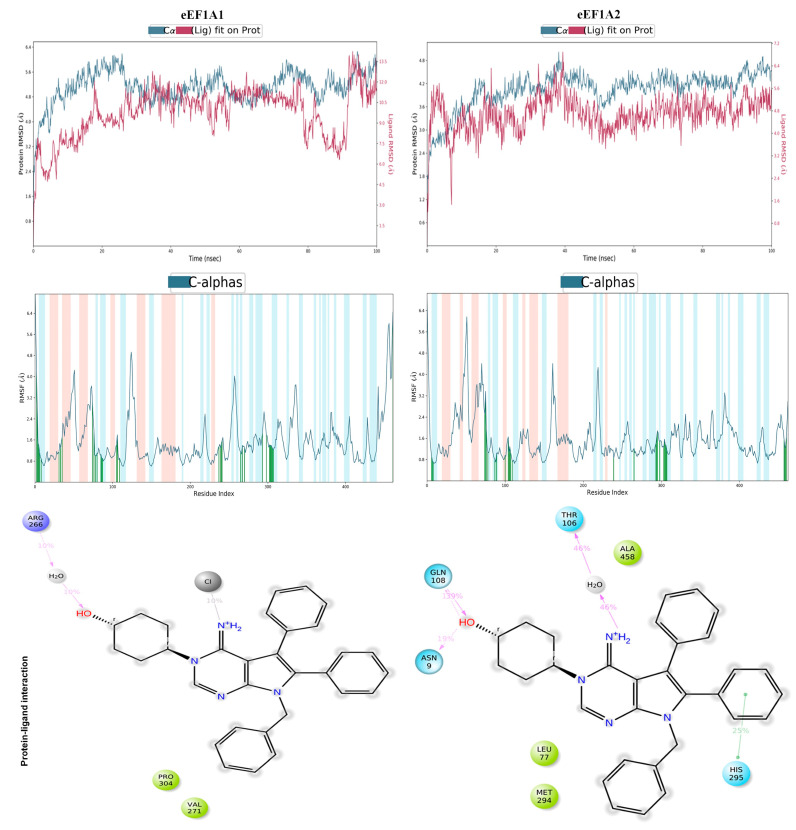
Summary of the RMSD, RMSF, and protein–ligand interactions of metarrestin-bound eEF1A1 and eEF1A2 proteins from MD simulations. MD simulations on the metarrestin-docked eEF1A1 and eEF1A2 complexes were carried out for 100 ns each in solvated environments (TIP3P water model, 0.15 M NaCl salt concentration) using the default protocol (NPT ensemble at temperature and pressure of 300 K and 1.01325 bar, respectively) in the Desmond tool (academic version) implemented in the Schrödinger suite using the Optimized Potentials for Liquid Simulations version 4 (OPLS4) force field. The analysis of the MD trajectory was carried out using the Simulation Interaction Diagram implemented in Maestro v11.2. The graphics were generated using PyMol [113].

Furthermore, to understand the stability of the modeled protein–ligand complexes, MD simulations were carried out for 100 ns each. Additionally, the analysis of the MD trajectory provided important information regarding the simulation convergence and the protein–ligand stability over time. Figure 4 presents the RMSD (root mean square deviation), RMSF (root mean square fluctuation), and interaction modes of metarrestin complexed with eEF1A1 and eEF1A2 proteins based on the 100 ns MD simulation.

The RMSD plots indicate the displacement of the protein (Cα atoms) and ligand (heavy atoms) relative to the reference frame over the simulation time. As seen, the MD simulations in both cases converged well over the simulation time. The RMSF plot indicates major fluctuations in the protein backbone over the simulation time. Major fluctuations in the RMSF plots are observed in residue positions such as Thr113-Thr116 (0.82–1.34 Å) and Leu250-Arg266 (0.99–1.14 Å) of the eEF1A1, while in eEF1A2, the fluctuations are observed at residue positions such as Ala46-Ala57 (2.24–1.66 Å).

Metarrestin formed indirect water-bridged hydrogen bonds with Arg266, ionic interaction with His7, and hydrophobic interactions with Val271 and Pro304 in eEF1A1. In the case of the eEF1A2–metarrestin complex, metarrestin formed direct hydrogen bonds with Gln108 and Asn109, water-mediated hydrogen bond interactions with Thr106, aromatic π-π stacking with His295, and hydrophobic interactions with Leu77, Met294, and Ala458. This further demonstrates that the eEF1A2–metarrestin complex forms much more stable contacts than the eEF1A1–metarrestin complex. Therefore, it can be concluded that metarrestin may inhibit eEF1A2 protein more efficiently than eEF1A1.

## 5. Metarrestin’s Pharmacokinetic/Pharmacodynamic (PK/PD) and Bioavailability in Animal Model

Rapid metabolism, early excretion, toxicity in normal healthy cells, coupled with relatively low bioavailability at tumor sites are major drawbacks associated with most biological agents, which limit their potential use as anticancer drugs [114,115,116]. Padilha et al. investigated the in vitro and in vivo metabolism and PK profiles of metarrestin [30]. The compound was administered intravenously and orally to a range of preclinical species, including mice, rats, dogs, pigs, and monkeys [30]. Metarrestin demonstrated excellent permeability and metabolic stability, which corresponded to its low clearance, substantial volume of distribution, and favorable oral bioavailability. Metarrestin also exhibited superior water solubility and permeability in the Parallel Artificial Membrane Permeability Assay, accompanied by a modest efflux ratio. These findings collectively highlight the promising PK profile of metarrestin across multiple species. It was also reported that metarrestin was metabolically stable in hepatocytes, liver microsomes, and S9 fractions from six species, including human samples [30]. Metarrestin exhibited minimal inhibition of key human CYP enzymes and did not exert significant inhibitory effects on critical human CYP enzymes, indicating a low potential for drug–drug interactions. The metabolism of metarrestin appears to be predominantly mediated by the CYP3A4 isoenzyme, with co-administration of other CYP3A4 substrates or inhibitors potentially influencing its pharmacokinetics, particularly its elimination. Additionally, the accumulation of metarrestin may be increased in individuals harboring genetic variations that affect CYP3A4 activity, potentially altering its clearance [117]. It revealed more than 80% oral bioavailability in all the species examined and concluded that metarrestin exhibits excellent absorption, distribution, metabolism, and excretion (ADME) and PK characteristics, making it suitable for clinical investigation [30].

Recent investigations have been conducted on the PK and pharmacodynamic (PD) of metarrestin in both wild-type C57BL/6 and the KPC transgenic pancreatic cancer mouse (Ras-driven Pdx1-Cre;LSL-Kras^G12D/+;^Tp53^R172H/+)^ model [29]. Metarrestin was administered via a single or repeated intravenous injections at varying doses (3 mg/kg, 10 mg/kg, and 25 mg/kg), by oral gavage (PO), or incorporated directly into the mice’s food (chow). The results demonstrated that metarrestin exhibited moderate plasma clearance and a high volume of distribution in mice, indicating favorable PK properties for systemic distribution [29]. Additionally, the oral bioavailability was greater than 80%, and there was significant dose-dependent normalization of FOXA1 and FOXO6 mRNA expression in KPC tumors [29]. Metarrestin can disturb desmoplastic stroma in pancreatic cancer, thus holding potential as a promising therapeutic option for pancreatic cancer [29]. Desmoplasia causes suboptimal drug delivery, alters the Tumor Microenvironment (TME), which includes tumor-surrounding blood vessels, fibroblasts, immune cells, extra cellular matrix, and other signaling molecules, and induces chemo-resistance in tumors [118,119].

Richardson et al. employed an FDA-approved bioanalytical technique to quantify metarrestin in human plasma for clinical PK applications [120]. They used ultra-high-performance liquid chromatography coupled with tandem mass spectrometry (uHPLC-MS/MS). The findings indicated that metarrestin demonstrated stability, with a degradation rate of ≤4.9% under various conditions. This approach was sensitive enough to quantify plasma concentrations from the lowest dosage cohort in the first-in-human Phase IA/B trial.

## 6. In Vitro and In Vivo Toxicity Studies of Metarrestin

A recent study assessed the safety, pharmacology, toxicology, and toxicokinetics of metarrestin using the beagle dog model [121]. Metarrestin was administered orally in capsule form every other day at doses of 0.25, 0.75, and 1.50 mg/kg/dose for 28 days. No fatalities were reported at the 1.5 mg/kg dose. However, clinical signs of toxicity were observed at mid- and high dose levels, including hypoactivity, salivation, tremors, ataxia, and intermittent seizure-like activity [121]. Additionally, treatment-related changes in food consumption and body weight were noted. The safety pharmacology study revealed no significant treatment-related effects on respiratory or physiological parameters, and no adverse histological changes were observed. Temporary thymic atrophy was detected, which was regarded non-detrimental. The study also determined the No-Observed-Adverse-Effect-Level (NOAEL) for metarrestin to be 0.25 mg/kg, which is a crucial factor for non-clinical risk assessment and for evaluating the potential for off-target effects [122]. On day 27, the mean Cmax for male and female dogs was 82.5 ng/mL, with a final AUC of 2521 hr ng/mL. In addition, Bourdi et al. evaluated metarrestin’s inhibitory effects on the hERG potassium channel in vitro. This study reported an IC_50_ of 0.13 µM for hERG inhibition, suggesting a potential for QT interval prolongation in clinical use. The safety margin for the cardiac arrhythmogenic effect was 4.1-fold (0.53 μM from the safety pharmacology study mean Cmax/0.13 μM in vitro hERG IC_50_) [121].

## 7. Cardiac Toxicity Prediction of Metarrestin

**Figure 5:**
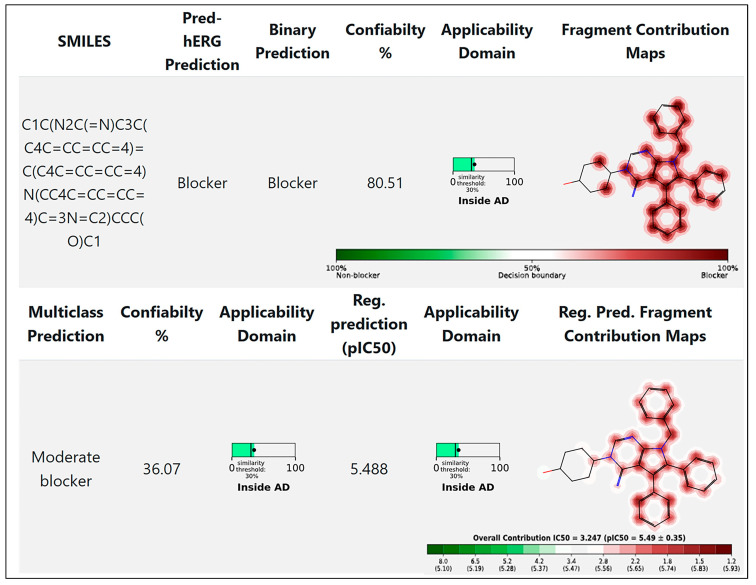
Cardiac toxicity of drugs derived from Pred-hERG in a map format for metarrestin. The cardiac toxicity of metarrestin was predicted by using the free online service Pred-hERG 5.0 (http://predherg.labmol.com.br/, accessed on 22 November 2024) [130].

The US FDA mandates testing of new bioactive compounds for their safety concerning the human ether-à-go-go-related gene (hERG). Non-cardiovascular drugs such as terfenadine [123], cisapride [124], and sertindole [125] have been withdrawn from the market due to their ability to block the hERG K^+^ channels, which can result in cardiac arrhythmia, and in severe cases, death [126]. Furthermore, the well-documented ligand promiscuity of this channel [127] has made hERG inhibition one of the most important targets to evaluate during the early stages of drug development. Binding affinity to the hERG K^+^ channel is measured experimentally using techniques like patch-clamp electrophysiology, the “gold standard” [128,129]. Our computational cardiac toxicity analysis (Figure 5), concluded using the Pred-hERG 5.0 software, indicates that metarrestin exhibits moderate hERG-blocking properties [130].

## 8. Clinical Trials

In early 2020, a unique first-in-human clinical trial (NCT04222413) was initiated to assess the safety and tolerability of metarrestin [131]. The study is titled “first-in-human phase I trial to investigate the safety, tolerability, pharmacokinetics, and biological and clinical activity of metarrestin in subjects with metastatic solid tumors” and is divided into two phases (Phase IA and Phase IB). The main objective of Phase IA is to determine the maximum tolerated dose (MTD) of metarrestin, and Phase IB is primarily designed to determine the objective response rate (ORR) according to evaluation criteria (RECIST 1.1) in patients treated with metarrestin at the MTD [131]. The first part of the Phase I trial intends to assess the safety of the compound in up to 24 adults with solid tumors of any type that have progressed after chemotherapeutic treatment over a 28-day treatment period. The trial plans to assess the effects of metarrestin on tumors in patients with metastatic breast cancer and to evaluate the compound’s safety and tolerability in pediatric cancer patients who are at least twelve years old, over a minimum two-month period.

## 9. Conclusions and Future Directions

PNC serves as a surrogate marker for cancer metastasis, leading to the development of targeted anticancer therapies. Preclinical studies indicate that metarrestin is an orally active, first-in-class, and specific PNC inhibitor. Preclinical investigations show that metarrestin is a very effective and selective inhibitor of PNC that may be taken orally. In vitro and in vivo, metarrestin interacts with the translation elongation factor eEF1A2 to alter the nucleolar structure and impede RNA polymerase I transcription. Furthermore, our molecular docking and MD simulation studies confirm that metarrestin binds to eEF1A1 and eEF1A2 with a strong and stable affinity and inhibits eEF1A2 more efficiently compared to eEF1A1. Metarrestin blocks metastatic development and extends survival in multiple cancer-type xenograft mouse models.

Metarrestin, which demonstrated promising antimetastatic activity across a range of solid cancers, has the potential to complement conventional therapeutic approaches, such as chemotherapy, surgery, or radiation therapy. This molecule can also be implemented in combination therapy, targeted therapy, radiopharmaceutical therapy (RPT), nanomedicine, and antibody–drug conjugates (ADCs). Combination therapy involves the concurrent use of multiple therapeutic modalities, such as chemotherapy, radiation therapy, immunotherapy, or surgical interventions to enhance treatment efficacy and overcome resistance mechanisms [132]. Studies on combination therapy using anticancer drugs and/or vaccines have gained considerable clinical success in cancer therapy in recent years [132,133]. Combining drugs can prevent resistance, enable the use of each drug at its optimal dose without intolerable side effects, and enhance response compared to using each drug alone. Moreover, nanoencapsulation may improve the poor water solubility of certain therapeutic compounds and can serve as a multi-drug delivery carrier system [134]. Solid tumors possess tortuous and poorly differentiated blood vessels (with 100–600 nm fenestrations), in comparison to healthy vasculature (1–2 nm fenestrations), enabling the extravasation of drug formulations with sizes of several hundred nanometers. Tumors have leaky blood vessels and poor lymphatic drainage, rendering them unable to remove extravasated nanomaterials. As a consequence, long-circulating nanoparticles (NPs) aggregate in tumors over time, a phenomenon known as the enhanced permeability and retention (EPR) effect [135,136]. In recent years, different types of NPs systems have emerged as a promising strategy for cancer treatment, including metastatic cancer [137,138,139,140,141]. Nanoparticles have many advantages over traditional cancer treatments, including targeted delivery, reduced toxicity, and improved efficacy [142]. Targeted therapy uses many different approaches, such as molecular targeted agents like small-molecule inhibitors [143,144], hormonal agents [145], immune checkpoint inhibitors, and targeted cytotoxic therapy [146,147]. Small-molecule inhibitors or antibodies that precisely inhibit signal transduction pathways play a crucial role in growth, proliferation, and survival [143,144,148]. Moreover, in ADC, a monoclonal antibody (mAb) is covalently attached to a cytotoxic drug via a chemical linker, enabling highly specific targeting and a potent killing effect for precise and effective cancer cell elimination [149,150,151]. Furthermore, RPT is emerging as a safe and effective targeted approach that involves the systemic or local delivery of radiation using pharmaceuticals that selectively bind to cancer cells or accumulate through physiological processes [152]. The potential to utilize metarrestin as a single drug, in combination therapies, or even in nanoformulations in therapeutic modalities of cancer treatment, will represent an exciting field with enormous potential to revolutionize cancer research in the years to come, warranting further investigation.

## Data Availability

Not applicable.
